# PYHIN1 regulates pro-inflammatory cytokine induction rather than innate immune DNA sensing in airway epithelial cells

**DOI:** 10.1074/jbc.RA119.011400

**Published:** 2020-02-26

**Authors:** Davide Massa, Marcin Baran, Jose A. Bengoechea, Andrew G. Bowie

**Affiliations:** ‡ School of Biochemistry and Immunology, Trinity Biomedical Sciences Institute, Trinity College Dublin, Dublin 2, Ireland; § Wellcome-Wolfson Institute for Experimental Medicine, Queen's University Belfast, Belfast, United Kingdom

**Keywords:** innate immunity, pattern recognition receptor (PRR), DNA viruses, interleukin 6 (IL-6), tumor necrosis factor (TNF), airway epithelial cells, cGAS, DNA sensing, PYHIN proteins, STING

## Abstract

Animal cells use pattern-recognition receptors (PRRs) to detect specific pathogens. Pathogen detection mounts an appropriate immune response, including interferon and cytokine induction. The intracellular PRR-signaling pathways that detect DNA viruses have been characterized, particularly in myeloid cells. In these pathways, cGMP-AMP synthase (cGAS) and the pyrin and HIN domain family member (PYHIN) protein interferon-γ–inducible protein 16 (IFI16) detect DNA and signal via stimulator of interferon genes protein (STING). However, although airway epithelial cells are frontline sentinels in detecting pathogens, information on how they respond to DNA viruses is limited, and the roles of PYHIN proteins in these cells are unknown. Here, we examined expression and activities of cGAS, STING, and PYHINs in human lung epithelial cells. A549 epithelial cells, commonly used for RNA-sensing studies, failed to respond to DNA because they lacked STING expression, and ectopic STING expression restored a cGAS-dependent DNA response in these cells. In contrast, NuLi-1 immortalized human bronchial epithelial cells did express STING, which was activated after DNA stimulation and mediated DNA-dependent gene induction. PYHIN1, which like IFI16 has been proposed to be a viral DNA sensor, was the only PYHIN protein expressed in both airway epithelial cell types. However, rather than having a role in DNA sensing, PYHIN1 induced proinflammatory cytokines in response to interleukin-1 (IL-1) or tumor necrosis factor α (TNFα) stimulation. Of note, PYHIN1, via its HIN domain, directly induced IL-6 and TNFα transcription, revealing that PYHIN proteins play a role in proinflammatory gene induction in airway epithelial cells.

## Introduction

Innate immune pattern recognition receptors (PRRs)^2^ have critical roles in sensing the presence of pathogens via detection of pathogen-associated molecular patterns (PAMPs), and of responding to cell and tissue damage by recognizing damage-associated molecular patterns. DNA-sensing PRRs have been intensively studied in recent years and have been shown to have an important role in alerting the cell to the presence of viruses and other pathogens by stimulating production of cytokines and interferons (IFNs), and also to drive autoimmunity by inappropriate responses to self DNA (1). For more than a decade, STING has been known to be a signaling adaptor protein with a role in DNA sensing (2, 3), and subsequently DNA PRRs were identified that activate STING. The best characterized of these is cGMP-AMP synthase (cGAS) (4). cGAS directly binds dsDNA, which stimulates the enzyme's catalytic activity leading to synthesis of the second messenger cGMP-AMP (cGAMP) (5). cGAMP then binds to STING dimers at the endoplasmic reticulum, causing an activating conformational change in STING. STING then translocates to perinuclear signaling compartments where it associates with and activates the kinase TBK1. TBK1 mediates activation of IRF3 and of other transcription factors downstream of STING, giving rise to IFN and cytokine induction (5). Consistent with the pathogenic role of dysregulated type I IFN production, human gain-of-function mutations in STING lead to systemic inflammation (6, 7), whereas excessive cGAS-STING signaling in response to either self or pathogen DNA is now linked to multiple diseases, including cancers (8).

Like cGAS, the human PYHIN (pyrin and hematopoietic interferon-inducible nuclear antigen with 200 amino acid repeats (HIN) domain) protein IFNγ–inducible protein 16 (IFI16) is also a PRR for dsDNA, acting upstream of STING to mediate cytokine and IFNβ induction (9). In human monocytes and keratinocytes, cGAS and IFI16 cooperate to activate STING, and all three proteins are required for a maximal response to DNA in these cells (10, 11). Although human airway epithelial cells are frontline sentinels in the detection of invading pathogens, in contrast to monocytes and keratinocytes, much less is known about how airway epithelial cells respond to DNA, and the role of cGAS, STING and PYHIN proteins in these cells. Thus, here we aimed to characterize the role of cGAS, STING, and PYHINs in human lung epithelial cell lines and their potential contribution to DNA sensing.

Apart from IFI16, there are four other human PYHIN proteins: absent in melanoma 2 (AIM2), myeloid cell nuclear differentiation antigen (MNDA), pyrin and HIN domain family member 1 (PYHIN1, also called IFIX), and pyrin domain only protein 3 (POP3) (12). PYHIN proteins are named because of the presence of an N-terminal pyrin domain, a motif that mediates oligomerization and is part of the death domain fold superfamily, and with the exception of POP3, one or two C-terminal HIN domains which are capable of binding dsDNA (12). Early studies with PYHIN proteins showed roles in cell cycle, tumor suppression, and transcriptional modulation, although the exact mechanisms whereby they mediated such functions remain unclear. Further, dysregulated PYHIN protein expression has been implicated in multiple autoimmune and inflammatory diseases (12–15). The first clear role for PYHINs in innate immunity came with the discovery that AIM2 is a cytosolic dsDNA sensor that mediates inflammasome activation after directly binding dsDNA via its HIN domain (16–18). AIM2 is now known to modulate protection against multiple bacteria, viruses, fungi, and protozoa (19). Next, IFI16 was shown to also be a PRR for dsDNA, mediating STING-dependent transcription factor activation and subsequent cytokine and IFN induction after HIN-dependent dsDNA recognition (9, 20). As well as acting as a PRR to sense certain viruses, IFI16 has also been shown to restrict replication and transcription of DNA viruses including herpes simplex virus-1 (HSV-1) in the nucleus by directly binding viral genomes (21–23), and also to suppress HIV-1 transcription by sequestering Sp1 (24). Compared with AIM2 and IFI16, very little is known about the role of MNDA and PYHIN1 in innate immunity.

The *PYHIN1* gene encodes up to six PYHIN1 protein isoforms because of alternative splicing, all six of which contain the pyrin domain, whereas only four contain a HIN domain (25). One of the longer isoforms, α1, was proposed as a tumor suppressor protein, being down-regulated in breast tumors and breast cancer cell lines (25). PYHIN1 isoforms interacted with and destabilized the oncoprotein HDM2, further attesting to a potential tumor suppressor role (26). PYHIN1 also suppressed cell invasion by up-regulation of the serine protease inhibitor and metastasis suppressor Maspin (27). A meta-analysis of genome-wide association studies correlated SNPs present in the PYHIN1 gene region with asthma in African-American and African-Caribbean populations, suggesting a possible link between this PYHIN protein and asthma pathogenesis (28). However, further analysis did not confirm the significant linkage between asthma-associated loci and PYHIN1 gene in individuals of African ancestry (29). Gene variants of PYHIN1 were also associated with pediatric inflammatory bowel disease (30). One study recently suggested that like IFI16, PYHIN1 may be a PRR for viral DNA, because knockdown of PYHIN1 expression in fibroblast cells, using shRNAs targeting all six isoforms, significantly enhanced HSV-1 titers compared with control cells, suggesting that PYHIN-1 inhibits HSV-1 replication (31). Further, PYHIN1 directly bound dsDNA via its HIN domain, and dsDNA transfection of HEK293 cells ectopically expressing PYHIN1 led to IFNβ mRNA induction (31). PYHIN1 was also recently shown to suppress HSV-1 viral gene expression in infected cells, and to be targeted by the virus for degradation (32).

To gain insights into the roles of STING, cGAS, and PYHIN proteins in human airway epithelial cells, we characterized the dsDNA sensing response in A549 cells, a commonly used human lung epithelial cell line for RNA virus studies. We also examined dsDNA sensing pathways for the first time in NuLi-1 cells, which are immortalized normal human bronchial epithelial cells. We found that A549 cells did not mount a robust innate immune response to dsDNA compared with RNA because of a lack of STING expression. Restoring STING expression in A549 cells led to a cGAS-dependent DNA response. In contrast, NuLi-1 cells did express STING, which was activated after DNA stimulation, and DNA-stimulated gene induction was STING-dependent. Both A549 and NuLi-1 cells expressed PYHIN1, and here we show for the first time a role for PYHIN1 not in DNA sensing, but in induction of pro-inflammatory cytokines (TNFα and IL-6).

## Results

### Lack of response of A549 cells to DNA viruses

**Figure 1.**
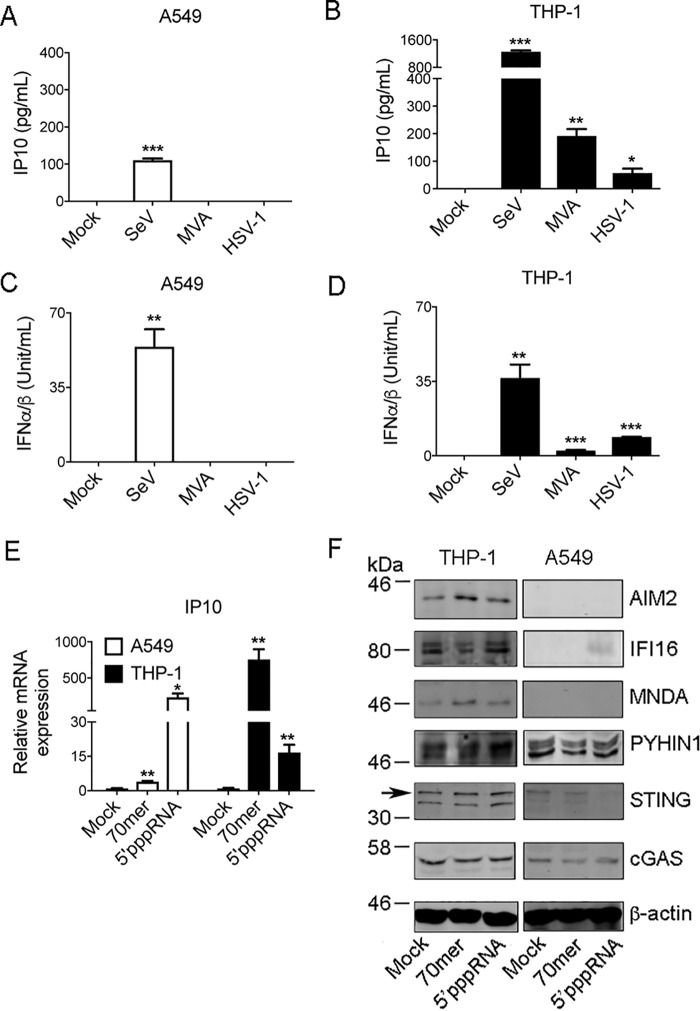
**Lack of innate immune response to DNA viruses in A549 cells compared with THP-1 cells.** *A–D*, 1 × 10^5^/ml A549 cells (*A* and *C*) or 1 × 10^6^/ml THP-1 cells pre-treated with 100 nm PMA for 24 h (*B* and *D*) were left untreated (mock) or infected with SeV, MVA, or HSV-1 for 24 h. IP10 in supernatants was measured by ELISA (*A* and *B*), whereas IFNα/β in supernatants was measured by bioassay (*C* and *D*). *E,* A549 or THP-1 cells were mock transfected (mock) or transfected with 2.5 μg/ml VACV dsDNA (70-mer) or 100 ng/ml 5′ppp-dsRNA for 6 h. IP10 mRNA was measured by qRT-PCR. Data are relative to mock untreated sample and normalized to β-actin levels. Data are mean ± S.D. of triplicate samples and are representative of three experiments (*A–E*). *F*, cells were stimulated as in *E* and lysed 24 h later. Lysates were immunoblotted with the indicated antibodies. *Arrow* indicates correct band for STING. Representative of three experiments. *, *p* < 0.05; **, *p* < 0.01; ***, *p* < 0.001 compared with untreated cells.

To examine innate immune DNA-sensing responses in airway epithelial cells we employed the human lung epithelial cell line A549, which is commonly used as an *in vitro* model for RNA virus infection of lung. We compared the response of A549 cells to human monocytic THP-1 cells differentiated with PMA, which are extensively characterized for cytosolic DNA sensing responses. Production of IP10 (CXCL10), a common marker of a PRR response, was assessed in both cells types after infection with RNA and DNA viruses. Surprisingly, although A549 cells responded robustly to the RNA virus Sendai (SeV), no IP10 production was apparent after infection of A549 cells with dsDNA viruses, namely the poxvirus modified vaccinia Ankara (MVA) or HSV-1 (Fig. 1*A*). Under the same infection conditions, THP-1 cells produced significant amounts of IP10 for each virus tested (Fig. 1*B*). The same trend was observed when type I IFN production was measured by bioassay: A549 cells only responded to SeV and not MVA or HSV-1, whereas type I IFN was elicited from THP-1 cells for all three viruses (Fig. 1, *C* and *D*). We next examined cellular responses to pure nucleic acid ligands. For this, a synthetic dsDNA known to stimulate STING-dependent responses, VACV 70-mer (9), and 5′triphosphate dsRNA (5′pppRNA), which is a ligand for the RNA sensing PRR RIG-I (33), were used. Transfection of the RIG-I ligand into either A549 or THP-1 cells induced robust *IP10* mRNA induction, whereas transfection of 70-mer caused substantial *IP10* induction in THP-1 cells but only a marginal response in A549 cells (Fig. 1*E*).

We wondered what would account for the difference in response to DNA and DNA viruses in A549 cells compared with THP-1 and so examined expression of cGAS, STING, and PYHIN proteins both in unstimulated and nucleic acid–stimulated cells by immunoblot. Interestingly, although cGAS expression was comparable in both cell types, STING expression was minimal to undetectable in A549 cells (Fig. 1*F*). We also noted that THP-1 cells expressed detectable amounts of the PYHINs IFI16, AIM2, MNDA, and PYHIN1, whereas A549 cells only expressed detectable PYHIN1 (Fig. 1*F*).

### STING expression restores a cGAS-dependent response to DNA in A549 cells

**Figure 2.**
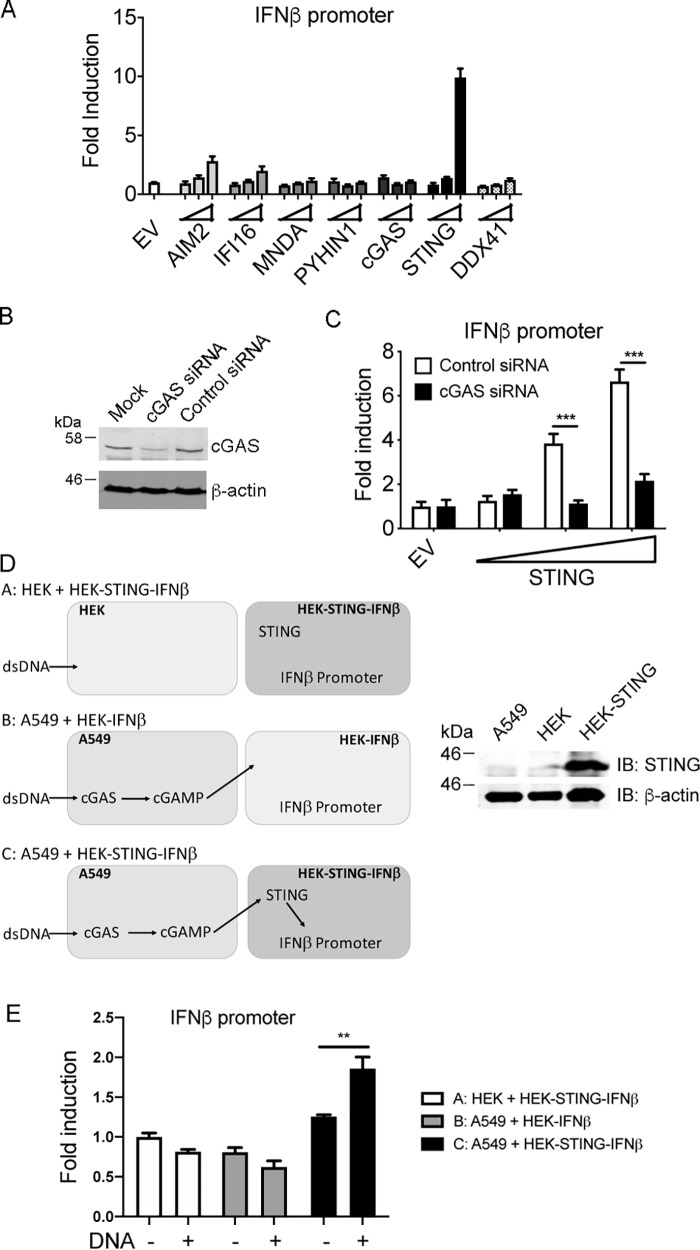
**STING expression restores a cGAS-dependent response to DNA in A549 cells.** *A,* 1 × 10^5^/ml A549 cells were transfected with IFNβ promoter luciferase reporter together with EV or increasing amounts of AIM2, IFI16, MNDA, PYHIN1, cGAS, STING, or DDX41 expression vectors. *Wedges* indicate increasing amount of the expression vectors (2, 10, 50 ng). Cells were lysed 24 h after transfection and assessed for reporter gene activity. Data are shown as relative fold-induction normalized to EV-only transfected cells. The data are mean ± S.D. of triplicate samples and are representative of three experiments. *B,* A549 cells (0.5 × 10^5^/ml) were transfected with 12.5 ng/ml control or cGAS siRNA 24 h and then again 48 h after cells were seeded. 24 h later cell lysates were generated and immunoblotted for cGAS and β-actin. Representative of two experiments. *C,* cells were treated with siRNA as in *B*, and 24 h later transfected with IFNβ promoter luciferase reporter together with EV or increasing amounts of STING expression vector. *Wedge* indicates increasing amount of STING expression vector (2, 10, 50 ng). Cells were lysed 24 h after transfection and assessed for reporter gene activity. ***, *p* < 0.001 compared with cells treated with control siRNA. *D*, co-culture cGAMP reporter assay. The schematic shows the three different co-cultures used in the assay involving A549 cells, HEK293T cells (HEK), HEK293T cells transiently expressing IFNβ promoter luciferase reporter (HEK-IFNβ), and HEK293T cells transiently expressing IFNβ promoter luciferase reporter and stably expressing STING (HEK-STING-IFNβ). Also shown is immunoblot confirming expressing of STING in HEK-STING cells but not HEK or A549 cells. *E*, cells co-cultured as shown in *D* were stimulated for 24 h with (+) or without (−) 2 μg/ml salmon sperm dsDNA. Cells were lysed and assessed for reporter gene activity. Data are shown as relative fold-induction normalized to unstimulated HEK: HEK-STING-IFNβ co-culture values. The data are mean ± S.D. of triplicate samples and representative of two experiments. **, *p* < 0.01 compared with cells not transfected with dsDNA.

We next determined whether transfection of plasmids encoding PYHINs, cGAS, STING, or DDX41 would restore an IFN response to DNA in A549 cells. Like cGAS and IFI16, DDX41 has been shown to be a PRR for dsDNA acting upstream of STING (34). In this reconstitution system, the transfected plasmid DNA provides the DNA PAMP, while the IFN response is measured by an IFNβ promoter reporter gene (10). This showed that transfection of a plasmid encoding STING, but not AIM2, IFI16, MNDA, cGAS, or DDX41, was sufficient to restore a robust IFNβ response (Fig. 2*A*). Fig. S1, *A–D*, confirms that all of the transfected plasmids expressed the expected proteins of correct molecular weight, albeit that some proteins, especially AIM2 were difficult to detect because of the low transfection efficiency of A549 cells compared with HEK293T cells (compare Fig. S1*A* to Fig. 6*C*).

Of the DNA PRRs that signal via STING (cGAS, IFI16, and DDX41), only cGAS expression was detectable in untransfected A549 cells (Fig. 1*A*), so we reasoned that ectopic STING expression was restoring a cGAS-dependent response. To test this, cGAS siRNA was employed, which effectively reduced endogenous cGAS expression (Fig. 2*B*). Fig. 2*C* shows that the IFNβ response restored by STING expression was cGAS-dependent, because cGAS siRNA completely prevented STING-stimulated IFNβ promoter induction. This suggested that A549 cells were capable of producing cGAMP in response to DNA, even though the IFNβ response was impaired because of lack of STING expression. To provide further evidence for this, we employed a cGAMP bioassay modified from a previous study (10). This assay is based on the fact that cGAMP expressed from one cell type can be detected by co-cultured HEK293T cells stably expressing STING (10, 35). Fig. 2*D* shows a schematic of the assay and confirmation of expression of STING in the HEK293T cells stably expressing STING, but not in normal HEK293T cells or A549 cells. Stimulation of A549 cells with dsDNA led to significant induction of IFNβ promoter activity in co-cultured HEK293T cells expressing STING, but not in co-cultured HEK293T cells not expressing STING (Fig. 2*E*). This indicates that after dsDNA stimulation A549 cells produce STING-stimulating activity, most probably cGAMP.

These result show that A549 cells had an impaired response to DNA because of lack of STING expression, and that delivery of STING to cells restored this response. Further, the restored response was completely cGAS-dependent.

### NuLi-1 cells do respond to DNA and DNA viruses and express STING

**Figure 3.**
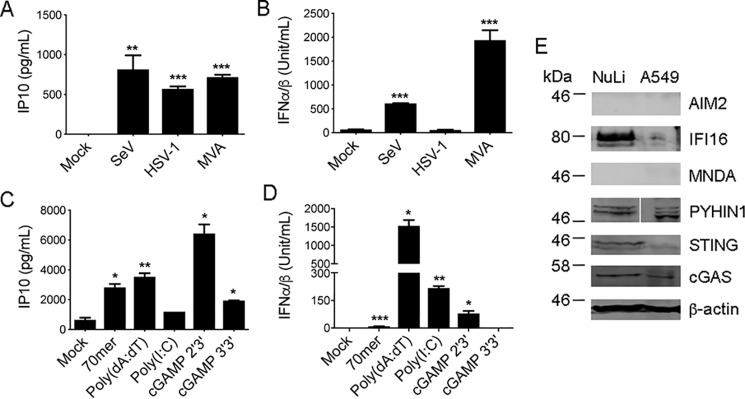
**The innate immune response to DNA is intact in NuLi-1 cells, which express STING.** *A–D,* 1.5 × 10^5^/ml NuLi-1 cells were left untreated (mock), or infected with SeV, MVA, or HSV-1 for 24 h (*A* and *B*). Alternatively, cells were mock transfected or transfected with 2.5 μg/ml VACV dsDNA 70-mer (70-mer), 2.5 μg/ml poly(dA-dT), 2.5 μg/ml poly(I:C) or 10 μg/ml cGAMP (10 μg/ml) for 24 h (*C* and *D*). IP10 in supernatants was measured by ELISA (*A* and *C*), whereas IFNα/β in supernatants was measured by bioassay (*B* and *D*). Data are mean ± S.D. of triplicate samples and are representative of three experiments. *, *p* < 0.05; **, *p* < 0.01; ***, *p* < 0.001 compared with untreated cells. *E,* NuLi-1 cells (1.5 × 10^5^/ml) or A549 cells (1 × 10^5^/ml) were lysed 24 h after seeding and lysates immunoblotted with the indicated antibodies. For PYHIN1 blot, *white vertical line* indicates where an irrelevant lane was spliced out of gel. Representative of three experiments.

Because A549 cells lacked STING expression and failed to respond robustly to DNA and DNA viruses, we wondered whether this was typical of human airway epithelial cells. Therefore, we also examined DNA responses in NuLi-1 cells, which are immortalized normal human bronchial epithelial cells, and which have not been previously assessed for RNA and DNA virus responses. Fig. 3*A* shows that in contrast to A549 cells, NuLi-1 cells infected with SeV, HSV-1, or MVA produced equally strong IP10 responses. Type I IFN production from NuLi-1 cells was also observed for SeV and MVA, although not for HSV-1 (Fig. 3*B*). The lack of a type I IFN response to HSV-1 in NuLi-1 cells is likely because HSV-1 encodes many proteins that antagonize type I IFN induction (36), rather than a lack of responsiveness of the dsDNA sensing pathway. Consistent with this, HSV-1 did induce IP10 in these cells, and MVA successfully elicited type I IFN from these cells. Because these cells have not been assessed for innate immune responses to nucleic acids previously, we also tested a range of pure DNA and RNA ligands, as well as cGAMP, a direct activator of STING. Transfection of DNA, RNA, and cGAMP all led to cytokine production, albeit that there were some differences between the IP10 and type I IFN response: Fig. 3, *C* and *D*, shows that 70-mer and poly(dA-dT) were equally potent at inducing IP10, whereas poly(dA-dT) gave a stronger type I IFN response than 70-mer. For dsRNA (poly(I:C), the type I IFN response was stronger than the IP10 response, whereas 2′3′ cGAMP stimulated both responses (Fig. 3, *C* and *D*). 2′3′ cGAMP is a stronger agonist of human STING than 3′3′ cGAMP (37, 38), and this was reflected in the weaker response elicited by the latter (Fig. 3, *C* and *D*). These results suggested that in contrast to A549 cells, NuLi-1 cells displayed STING-dependent responses. Consistent with this, immunoblotting showed easily detectable expression of STING in NuLi-1 cells compared with A549 (Fig. 3*E*). Like A549 cells, NuLi-1 also expressed cGAS and PYHIN1, and in contrast to A549 cells, NuLi-1 also showed robust IFI16 expression. Thus, immortalized normal human bronchial epithelial cells respond to both DNA and RNA, and express STING.

### STING activation in NuLi-1 cells controls the DNA response

**Figure 4.**
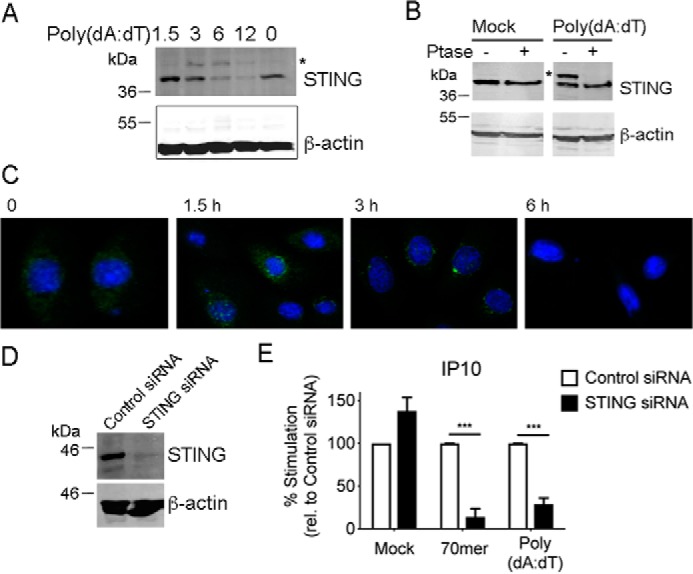
**STING activation in NuLi-1 cells controls the innate immune response to DNA.** *A,* NuLi-1 cells (1.5 × 10^5^/ml) were transfected with 2.5 μg/ml poly(dA-dT) for the indicted times (h). Cells were then lysed and lysates immunoblotted with the indicated antibodies. Representative of three experiments. *B*, cells were mock transfected (mock) or stimulated with poly(dA-dT) for 3 h and then lysed. Lysates were incubated at 37 °C for 45 min with (+) or without (−) the addition of λ protein phosphatase (Ptase), and then immunoblotted with the indicated antibodies. Representative of two experiments. In *A* and *B*, the asterisk indicates phosphorylated STING as determined by phosphatase treatment in *B. C,* cells were seeded on coverslips in 12-well plates and grown overnight at 37 °C. Cells were then transfected with 2.5 μg/ml poly(dA-dT) for the times indicated, and fixed and stained for confocal microscopy analysis. STING protein is visualized in *green* and *blue* represents DAPI-stained cell nuclei. Images are representative of three separate pictures taken in different locations of the same coverslip. *D,* cells (0.75 × 10^5^/ml) were transfected with 20 μm/ml control or STING siRNA 24 h and then again 48 h after cells were seeded. 24 h later cells were mock transfected for 24 h and then lysates were generated and immunoblotted for STING and β-actin. Representative of two experiments. *E,* cells were treated as in *D* and either mock transfected (mock) or transfected with 1 μg/ml VACV dsDNA 70-mer (70-mer) or poly(dA-dT) for 24 h. IP10 in supernatants was measured by ELISA. Data are mean ± S.E. of three experiments and expressed as a percentage of the IP10 response for control siRNA. *, *p* < 0.05; **, *p* < 0.01; ***, *p* < 0.001 compared with cells treated with control siRNA.

Because of the paucity of knowledge about STING activation in human airway epithelial cells, and the fact that NuLi-1 cells responded to DNA and expressed STING, we examined the role of STING in the DNA response of these cells in detail. Transfection of cells with dsDNA caused a time-dependent phosphorylation of STING, which is a hallmark of activation of the STING pathway (39). This was measured by the appearance of an upper band in the STING immunoblot peaking at 3–6 h post DNA transfection (Fig. 4*A*), which was sensitive to phosphatase treatment (Fig. 4*B*). Degradation of STING after phosphorylation was also observed, as seen by reduced detection of STING by immunoblot from 6–12 h (Fig. 4*A*). STING degradation is a hallmark of STING activation in some cell types (40). STING activation was also confirmed by confocal microscopy, which showed the characteristic movement and clustering of STING to the perinuclear region (3) after 1.5 h of DNA transfection, and then reduced detection after 6 h because of STING degradation (Fig. 4*C*). To test whether STING activation was required for DNA sensing in NuLi-1 cells, siRNA targeting STING was utilized. Targeting STING by siRNA effectively reduced endogenous STING expression (Fig. 4*D*) and led to potent inhibition of both 70-mer– and poly(dA-dT)–stimulated IP10 production (Fig. 4*E*). These data show that, in contrast to A549 cells, the STING signaling pathway operates in normal human bronchial epithelial cells and is required for innate immune DNA sensing. Also, innate immune sensing of DNA in airway epithelial cells correlates with STING expression, rather than with PYHIN protein expression.

### PYHIN1 is required for pro-inflammatory cytokine production in airway epithelial cells

**Figure 5.**
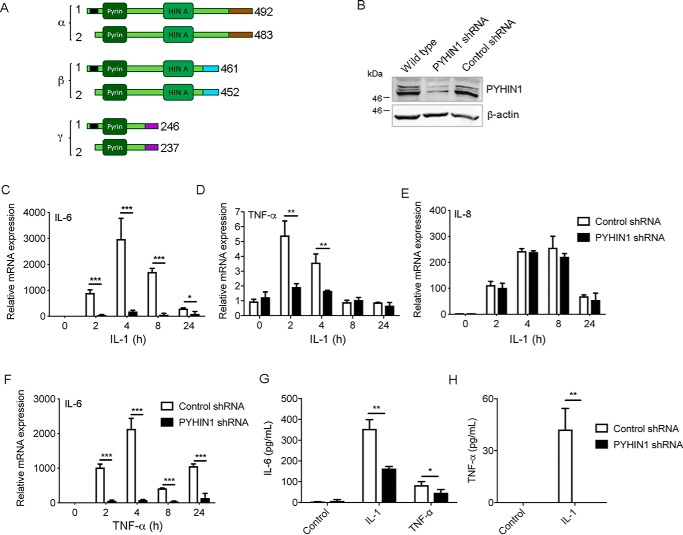
**PYHIN1 is required for cytokine production in A549 cells.** *A,* schematic of six protein isoforms of PYHIN1. Color coding is as follows: *black*, region of nine amino acids absent from isoforms α2, β2 and γ2; *brown*, region unique to α forms; *cyan*, region unique to β forms; *magenta*, region unique to γ forms. *B,* 0.25 × 10^5^/ml A549 cells were transduced for 72 h with lentivirus expressing control shRNA or shRNA targeting all six PYHIN1 isoforms. Cell were then harvested and lysates immunoblotted for PYHIN1 and β-actin. Representative of two experiments. *C–E,* cells were treated as in *B*, and then stimulated with 20 ng/ml IL-1 for the indicated times. IL-6 (*C*), TNFα (*D*), and IL-8 (*E*) mRNA was measured by qRT-PCR. *F,* as per *C*, except cells were stimulated with 100 ng/ml TNFα rather than IL-1. For *C–F*, data are relative to untreated cells and normalized to β-actin levels. *G* and *H,* cells were stimulated with IL-1 or TNFα for 24 h and IL-1 (*G*) or TNFα (*H*) measured in supernatants by ELISA. Data are mean ± S.D. of triplicate samples and are representative of three experiments. *, *p* < 0.05; **, *p* < 0.01; ***, *p* < 0.001 compared with cells treated with control siRNA.

Because cGAS and STING seemed to fully account for the DNA-sensing response in airway epithelial cells (Figs. 2*C* and 4*E*), we wondered what role PYHIN proteins might play in this cell type. We noted that PYHIN1 was strongly expressed in both A549 and NuLi-1 cells (Fig. 3*E*), and indeed was the only PYHIN protein detected by immunoblot in A549 cells (Fig. 1*F*). This provided an opportunity to assess the role of PYHIN1 in A549 cells in the absence of other PYHINs, which was important because PYHINs might have redundant effects and compensate for the loss of each other. Therefore we used shRNA targeting PYHIN1 in A549 cells to screen for PYHIN1-dependent cellular responses. PYHIN1 can be expressed as six different protein isoforms (25) as shown in Fig. 5*A*, which are commonly detected as a triplet of bands by immunoblot (Fig. 5*B*). A549 cells were transduced with lentivirus expressing control shRNA, or shRNA targeting all six isoforms of PYHIN1 which was effective at suppressing PYHIN1 expression (Fig. 5*B*). Apart from roles in DNA sensing, some specific mouse and human PYHINs have been shown to directly regulate gene induction in the nucleus, in specific cell types, independent of the stimulus used (41, 42). Because airway epithelial cells are responsive to pro-inflammatory cytokines, as an alternative to nucleic acid stimulation, we examined gene induction after stimulation of A549 cells with a physiologically relevant stimulus, the pro-inflammatory cytokine IL-1, to assess effects of PYHIN1 on gene induction independent of any DNA-sensing role. Treatment of A549 cells with IL-1 elicited time-dependent induction of IL-6, TNFα and IL-8 mRNA (Fig. 5, *C–E*). To our surprise, PYHIN1 shRNA significantly and potently inhibited both IL-6 and TNFα mRNA induction but had no effect on IL-8 (Fig. 5, *C–E*). The requirement of PYHIN1 for IL-6 mRNA induction was independent of the stimulus used, because TNFα-stimulated IL-6 mRNA was also almost completely prevented in cells expressing PYHIN1 shRNA (Fig. 5*F*). Consistent with the requirement for PYHIN1 for IL-6 and TNFα mRNA induction, secretion of IL-6 in response to IL-1 or TNFα, and secretion of TNFα in response to IL-1, were significantly impaired by PYHIN1 shRNA (Fig. 5, *G* and *H*). Thus, PYHIN1 is required for both IL-6 and TNFα production in A549 cells.

### PYHIN1 regulates the IL-6 and TNFα gene promoters

**Figure 6.**
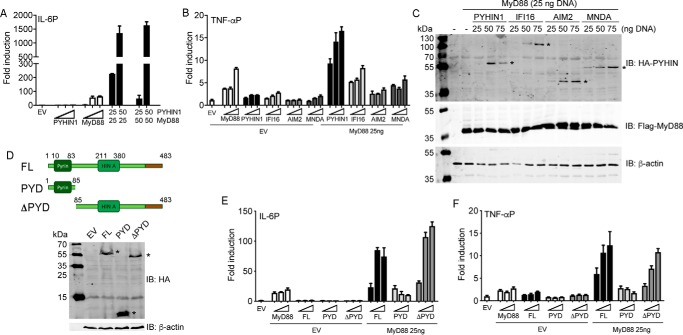
**PYHIN1 regulates the IL-6 and TNF promoters.** *A,* 1.5 × 10^5^/ml HEK293T cells were transfected with IL-6 promoter luciferase reporter together with empty vector (EV) or PYHIN1-α2 and MyD88 expression vectors (ng). *Wedges* indicate increasing amount of the expression vectors (25, 50, 75 ng). *B,* cells were transfected with TNFα promoter luciferase reporter together with EV or PYHIN and MyD88 expression vectors. *Wedges* indicate increasing amount of the expression vectors (25, 50, 75 ng). *C*, cells from (*B*) transfected with MyD88 and increasing amount of PHYINs were lysed 24 h after transfection and assessed for protein expression by immunoblotting using anti-HA Ab to detect PYHINs, anti-FLAG Ab to detect MyD88 and anti–β-actin Ab as a protein loading control. *Asterisk* (*) indicates expression of PYHIN protein at the correct molecular weight. *D,* schematic of PYHIN1-α2 truncation constructs used: full-length PYHIN1-α2 (*FL*), pyrin domain only (PYD) and PYHIN1-α2 lacking the pyrin domain (ΔPYD). To confirm expression of truncations, HEK293T cells were seeded in 6-well plates (4 × 10^5^ cells) and transfected with 2 μg of empty vector (EV), FL, PYD, or ΔPYD. Cells were lysed 24 h later with assessed for protein expression using anti-HA Ab to detect PYHIN1 constructs and anti–β-actin Ab as a protein loading control. *Asterisk* (*) indicates expression of PYHIN1 constructs at the correct molecular weight. *E* and *F,* cells were transfected with IL-6 (*D*) or TNFα (*E*) promoter luciferase reporter together with the expression vectors indicated, prior to reporter gene analysis. In each panel, *wedges* indicate increasing amount of the expression vectors (25, 50, 75 ng). Cells were lysed 24 h after transfection and assessed for reporter gene activity. Data are shown as relative fold-induction normalized to EV only transfected cells. The data are mean ± S.D. of triplicate samples and are representative of three experiments.

We next wondered how PYHIN1 was regulating IL-6 and TNFα mRNA induction. Because nucleic acid sensing would not be involved in pro-inflammatory cytokine-stimulated mRNA induction, we reasoned that PYHIN1 may directly regulate promoter induction of IL-6 and TNFα. This would be consistent with the fact that another PYHIN protein, IFI16, has been shown to directly modulate both viral and host gene promoters in certain circumstances (42, 43), and with the fact that, like IFI16, PYHIN1 can be detected in the nucleus (31, 32). Furthermore, the fact that PYHIN1 was required for both IL-1– and TNFα-mediated IL-6 induction (Fig. 5, *C*, *F*, and *G*) was suggestive of a gene promoter proximal role. To test whether PYHIN1 had a direct effect on cytokine promoters, an IL-6 promoter reporter gene assay was used in HEK293T cells. Transfection of cells with a plasmid encoding MyD88, an adaptor protein for IL-1 signaling, led to IL-6 promoter induction (Fig. 6*A*). Interestingly, although transfection of the PYHIN1 α2 isoform alone had no effect on the IL-6 promoter, it had a potent synergistic effect with MyD88 on IL-6 promoter induction (Fig. 6*A*). Further, PYHIN1 also synergized with MyD88 for TNFα promoter induction, which was not seen for other PYHIN proteins (IFI16, AIM2 and MNDA, Fig. 6*B*), even though all four PYHIN proteins were detectably expressed in the assay when combined with MyD88 (Fig. 6*C*, *upper panel*), and expression of PYHINs did not affect MyD88 expression (Fig. 6*C*, *middle panel*). To test which PYHIN1 domains are involved in cytokine promoter induction, we generated truncated proteins, and confirmed their expression (Fig. 6*D*). For IFI16 and AIM2, the pyrin domain is required for protein oligomerization after DNA sensing (44, 45). In contrast, here we found that the PYHIN1 pyrin domain was dispensable for PYHIN1-dependent cytokine gene promoter induction, because like the full-length protein, a truncated PYHIN1 lacking the N-terminal pyrin domain also synergized with MyD88 for both IL-6 and TNFα promoter induction, whereas the pyrin domain alone had no effect (Fig. 6, *E* and *F*).

Together, these data show a novel role for PYHIN1 in cytokine gene induction, rather than DNA sensing, in human airway epithelial cells.

## Discussion

In recent years, extensive progress has been made in understanding how DNA and DNA viruses are sensed by intracellular PRRs including cGAS and PYHIN proteins such as IFI16. Although the lung is a key point of entry for pathogens, less is known about how airway epithelial cells respond to PAMPs compared with knowledge on myeloid cells and other cell types, and PYHIN proteins are yet to be prescribed a role in these cells. Therefore we sought to better understand how cGAS, STING, and PYHINs function in human airway epithelial cells, and began by examining DNA sensing pathways in A549 cells, because they are very commonly used to model RNA virus infections *in vitro*. Unexpectedly, A549 cells exhibited minimal to no response to DNA and DNA viruses compared with RNA and RNA viruses, and we showed that this was primarily because of a lack of STING expression. Consistent with this, the DNA response was rescued by delivery of STING to A549 cells, and this rescued response was completely cGAS-dependent, as shown by siRNA.

The question then became whether this lack of response to DNA in A549 cells accurately represented airway epithelial cells in general, because A549 cells were derived from a lung carcinoma (46), and STING expression has been shown to be suppressed in some cancer and tumor cell lines (47). To address this, we turned to NuLi-1 cells, which are normal human lung bronchial epithelial cells immortalized by dual retroviral infection with HPV-16E6/E7-LXSN and hTERT-LXSN (48, 49). NuLi-1 cells have been used as an *in vitro* model for the epithelial barrier of the lower respiratory tract in different studies, based on the development of cystic fibrosis and the effect of cigarette smoke on the airway system (50–52). The response to viruses and PAMPs has not yet been well-characterized in these cells, although they have been shown to express TLRs and respond to TLR ligands in a manner similar to primary human nasal epithelial cells (53). Here we found that NuLi-1 cells did respond to DNA and DNA viruses, and also the direct STING ligand cGAMP. Consistent with this, and in contrast to A549 cells, they expressed detectable amounts of STING protein. We then characterized for the first time an active STING response in NuLi-1 cells, because poly(dA-dT) stimulation caused STING phosphorylation and degradation, and also STING movement to and clustering at a perinuclear location. These responses are hallmarks of activation of the STING signaling pathway in cells where DNA sensing is well-characterized, although STING degradation after activation does seem to be specific to only certain cell types (40). Importantly, STING siRNA in NuLi-1 cells potently inhibited DNA-stimulated chemokine production. These data show that NuLi-1 cells are likely to be a good model for DNA sensing studies in the human airway. Overall, the results showed that the response of human airway epithelial cells to DNA strongly correlates with STING expression.

Until recently it was unclear why STING expression is repressed in A549 cells. We sequenced the proximal genomic region of the STING promoter in A549 cells and did not observe any major sequence differences or deletions compared with THP-1 cells (data not shown), suggesting that the STING gene is intact in A549. However Olagnier *et al.* (54) have now provided an explanation for suppressed STING protein expression in A549 because they showed that the transcription factor NRF2 (nuclear factor (erythroid-derived 2)-like 2) can suppress STING expression, and that NRF2 is constitutively active in A549 cells. When they treated A549 cells with NRF2 siRNA, both STING protein expression and DNA responses were restored (54). In normal cells, including the airway epithelial, KEAP1 (Kelch-like ECH-associated protein 1) negatively regulates NRF2. However in at least some lung cancer cell lines, including A549, KEAP1 mutations abolish KEAP1 repressor activity leading to elevated NRF2 (55). Thus other lung cancer cell lines are also likely to display unnaturally low expression of STING. Other ways that STING has been shown to be silenced in human cancer and tumor cell lines include by hypermethylation of the STING promoter and by mutations in STING or cGAS leading to loss of function or loss of protein expression (56). These studies are consistent with the idea that because the cGAS-STING–induced immune response has anti-tumor effects (for example via IFN induction), cancer cells, like viruses, must evade DNA-sensing responses to persist (47). Thus, like A549 cells, many cell lines derived from cancers will not be useful to model STING-dependent DNA-sensing pathways *in vitro*. In comparison, as has been noted, RNA sensing pathways in cancer cell lines are significantly less affected (57).

We found that PYHIN1 was expressed in both A549 and NuLi-1 cells. The lack of a DNA-sensing response in A549 together with the restricted repertoire of PYHIN expression allowed us to examine whether PYHIN1 had functions other than DNA sensing, and also to explore PYHIN protein function for the first time in human airway epithelial cells. Because A549 cells did not express other PYHINs, PYHIN1 function could be examined using RNAi without a concern that other PYHINs would compensate for PYHIN1 loss because of functional redundancy. This led to an important new insight into PYHIN1 function, namely that it was required for optimal IL-6 and TNFα transcriptional induction after cytokine stimulation of cells. Hence, similar to IFI16, PYHIN1 has now been implicated in DNA sensing (31), viral restriction (32), and host immune gene regulation (this paper). IFI16 was previously shown to be required for maximal promoter induction of type I IFNs and IFN-stimulated genes, but not IL-6, in human THP-1 monocytes (42). Here we showed a role for PYHIN1 in maximal promoter induction of IL-6 and TNFα in A549 cells. The basis for these cell- and promoter-specific functions of different human PYHINs is currently unclear but likely to be accounted for in part by different amounts of PYHIN expression in different cell types, as attested to by the human proteome map (58).

How exactly PYHIN1 regulates pro-inflammatory cytokine promoters is not known. That PYHIN1 is mainly expressed in the nucleus, and that PYHIN1 was required for both IL-1– and TNFα-mediated IL-6 induction, but not IL-8 induction, is suggestive of a gene promoter proximal role. This hypothesis is consistent with the ability of ectopically expressed PYHIN1 α2 isoform to directly boost MyD88-dependent IL-6 and TNFα-promoter reporter genes. Given the similar domain structure, PYHIN1 might be expected to regulate transcription in a manner similar to IFI16. However more is known about how IFI16 suppresses viral transcription, namely by promoting the addition of heterochromatin marks (59, 60) or by sequestering Sp1 away from viral promoters (21, 24), compared with what is known about how IFI16 might enhance host transcription. On the latter, IFI16 positively affects host transcription by activating p53, and Liao *et al.* (61) showed that the two HIN domains of IFI16 directly interact with p53 and enhance p53-DNA complex formation and transcriptional activation. Because we found that for PYHIN1, a fragment containing the HIN domain but not the pyrin domain activated the IL-6 and TNFα promoters to a similar degree to full-length IFI16, a similar mechanism may operate here, whereby PYHIN1 engages with a transcription factor via its HIN domain, to boost the efficiency of transcription factors in stimulating transcription. Ectopically expressed PYHIN1 has been shown to associate with proteins involved in chromatin remodeling and transcription, for example proteins of the five friends of methylated chromatin (5FMC) complex which desumoylate target transcription factors (31). However future studies will need to determine what candidate proteins PYHIN1 interacts with that might be involved specifically in IL-6 and TNFα but not IL-8 promoter induction, or indeed whether PYHIN1 directly binds to these promoters.

All together this and other recent studies on the PYHIN proteins confirm their functional diversity in innate immunity. Overall human PYHINs seem to function remarkably similar to the nuclear oligomerization domain (NOD) family of proteins in that some are inflammasome activators (AIM2 and NOD NLRP3) (18, 62), some are PRRs (IFI16 and NOD NOD1) (9, 63), and some are transcriptional regulators (PYHIN-1 and NODs NLRP12 and CIITA) (64, 65). To our knowledge, this study is the first to link PYHIN function to pro-inflammatory cytokine gene induction, and the first demonstration of a role for PYHIN proteins in human airway epithelial cells.

## Experimental procedures

### Cell culture

THP-1 human monocytic cells were maintained in RPMI containing 10% (v/v) fetal calf serum, 2 mm l-glutamine and 10 μg/ml ciprofloxacin. THP-1 cells were differentiated by treatment with 100 nm PMA for 24 h. The A549 human lung epithelial cell line and HEK293T cells were maintained in Dulbecco's modified Eagle's medium containing 10% (v/v) fetal calf serum and 10 μg/ml ciprofloxacin. NuLi-1 cells, which are immortalized normal human bronchial epithelial cells, were grown in Bronchial Epithelial Cell Growth Basal Medium^TM^ (Lonza) supplemented with Bronchial Epithelial Cell Growth Medium SingleQuots (Lonza), 10 μg/ml of ciprofloxacin, in flasks pre-coated with Human Placental Collagen Type IV (Sigma-Aldrich).

### Viruses and cell stimulants

HSV-1 was a gift from S. Paludan (Aarhus University, Denmark). MVA was a gift from I. Drexler (Düsseldorf University, Germany). SeV Cantell strain was from ECACC. Serial dilutions of SeV indicated the optimal dilution to elicit IFN without causing cell death as 1:200, which was used in all experiments. All other viruses were used at a multiplicity of infection of 10. Cell stimulants used were 70-mer dsDNA (70-mer) (9); poly(dA-dT), poly(I:C) (both Sigma-Aldrich); cGAMP and 5′pppRNA (both InvivoGen). 70-mer, poly(dA-dT), poly(I:C), 5′pppRNA, and cGAMP were delivered to cells by transfection using Lipofectamine^®^2000 (Invitrogen), at the concentrations indicated in figure legends. IL-1α (PBL Interferon Source) and TNFα (PeproTech) were also used.

### ELISA

Cell culture supernatants were assayed for the presence of IP10 (CXCL10), TNFα, IL-6, and IL-8 by ELISA kits (R&D Systems).

### Type I IFN bioassay

Bioactive type I IFNs in the supernatants were detected using HEK-Blue IFNα/β bioassay (InvivoGen). Supernatants and IFNα standard were diluted in the test medium (DMEM, 10% FCS, 50 units/ml penicillin, 50 μg/ml streptomycin, 100 μg/ml normocin). HEK-Blue IFNα/β cells (2.8 × 10^5^ cells/ml) were seeded to 96-well plate containing supernatant, standard and blank. Following 24-h incubation at 37 °C, secreted SEAP was detected by QUANTI-Blue and absorbance was measured at 620 nm.

### Quantitative RT-PCR

mRNA expression was analyzed by RT-PCR. The mRNA expression levels were normalized to β-actin mRNA and the relative quantification values were calculated. The mean relative quantification values calculated from three corresponding replicates was presented as fold induction relative to the control sample set to 1. *Error bars* represent the S.D. calculated using GraphPad Prism software. Primers used were: β-actin, forward, 5′-CGCGAGAGAAGATGACCCAGATC-3′ and reverse, 5′-GCCAGAGGCGTACAGGGATA-3′; IP10, forward, 5′-GGCAATCAAGGAGTACCTCTCT-3′ and reverse, 5′-GCAATGATCTCAACACGTGGAC-3′; IL-6, forward, 5′-ACCCCCAGGAGAAGATTCCA-3′ and reverse, 5′-CACCAGGCAAGTCTCCTCATT-3′; TNFα, forward, 5′-GAACCCCGAGTGACAAGCCTG-3′ and reverse, 5′-TCAGCTCCACGCCATTGGCCA-3′; IL-8, forward, 5′-CTCTGTGTGAAGGTGCAGTTTTG-3′ and reverse, 5′-AAGCTTTACAATAATTTCTGTGGT-3′.

### Immunoblotting

Antibodies used were anti-AIM2 (mouse 3B10, AdipoGen Life Sciences), anti-IFI16 (mouse 1G7, Santa Cruz Biotechnology), anti-MNDA (mouse 3C1, Cell Signaling Technology), anti-PYHIN1 (a gift from Y. Ding, UTMD Anderson Cancer Center, Houston, TX), anti-STING (rabbit D2P2F, Cell Signaling Technology), anti-cGAS (rabbit, Sigma-Aldrich), anti-HA (Covance), anti-FLAG (Sigma), anti-Myc (Sigma), and anti–β-actin (mouse AC-74, Sigma-Aldrich). After cell stimulation or treatment with siRNAs, lysates were prepared from 96-well plates or 6-well plates and proteins separated by SDS-PAGE. Resolved proteins were transferred to PVDF membranes and immunoblotted with the indicated antibodies.

### Reporter gene assays

Plasmids used in reporter gene assays were as follows: pCMV-HA (Clontech), which served as an empty vector (EV) control in experiments, was used to create expression vectors for AIM2, IFI16, MNDA, STING, PYHIN1-α2, whereas FLAG-MyD88, FLAG-cGAS, and Myc-DDX41 were also used. Truncations of PYHIN1 were cloned from PYHIN1-α2: PYD comprised amino acids 1–85, whereas ΔPYD comprised amino acids 85–483. The following luciferase promoter reporter genes were used: IFNβ promoter (gift from T. Taniguchi, University of Tokyo, Japan), TNFα promoter (gift from I. Udalova, Imperial College London, UK), and IL-6 promoter (gift from M. Wewers, Ohio State University). HEK293T (1.5 × 10^5^ cell/ml) or A549 (1 × 10^5^ cell/ml) cells were seeded in 96-well plates and transfected 24 h later with expression vectors and 60 ng/well luciferase reporter genes as indicated in figure legends, using GeneJuice^®^. In all cases, 20 ng/well of pGL3 *Renilla* reporter gene (Promega) was cotransfected to normalize data for transfection efficiency. The total amount of DNA transfected was kept constantly at 230 ng by adding EV. Cells were lysed in Passive Lysis Buffer (Promega), and whole cell lysates were analyzed for luciferase activity. Firefly luciferase activity was normalized to Renilla luciferase activity, and data are expressed as the mean fold induction, relative to control levels, for a representative experiment performed in triplicate.

For the cGAMP reporter assay, normal HEK293T cells or HEK293T cells stably expressing STING (35) were seeded in 10-cm dishes at 2 × 10^5^ cells/ml and incubated overnight at 37 °C. Cells were transfected 24 h later with 8 μg IFNβ-luc reporter plasmid using GeneJuice^®^. After 6 h, the medium was removed and cells were harvested in fresh DMEM, counted and co-seeded at a 1:1 ratio into 24-well plates with either A549 cells or untransfected HEK293T cells at total cell concentration of 1.5 × 10^5^ cells/ml. Cells were stimulated 24 h later by transfection with 2 μg/ml of salmon sperm DNA (Invitrogen) using Lipofectamine 2000. 24 h later cells were harvested for luciferase reporter gene analysis as described above.

### RNAi

Lipofectamine^®^2000 was used to transfect cells with siRNA. A549 and NuLi-1 cells were seeded in 12- or 24-well plates, respectively. siRNA silencing was performed by addition of siRNA both 24 and 48 h after seeding the cells. Cells were then analyzed 24 h later. siRNAs (Qiagen) used were as follows: Control, 5′-AATTCTCCGAACGTGTCACGT-3′; cGAS, 5′-AAGGAAGGAAATGGTTTCCAA-3′; and STING, 5′-TGGCATCAAGGATCGGGTTTA-3′.

To generate A549 cells stably expressing PYHIN1 shRNA, shRNA encoding lentivirus was first generated in HEK293T cells. An shRNA hairpin sequence designed to target all six isoforms of PYHIN1 (5′-ATTCTAGGAAATGGAGTATTA-3′ target sequence) or control shRNA was cloned into a pLKO.1 lentiviral expression vector. HEK293T cells in 10-cm dishes were transfected with 4 μg pLKO.1, 3 μg psPAX2 packaging plasmid and 1 μg pMD2.G envelope plasmid. Supernatants were harvested 48 h later and replaced with fresh media. The lentivirus-containing supernatants were centrifuged and then filtered through 0.45-μm filters. They were stored at 4 °C until use. The supernatants were harvested again at 72 h, centrifuged, and filtered. A549 cells were infected with 10% (v/v) lentivirus-containing supernatants together with 8 μg/ml polybrene for 24 h. The medium was discarded and replaced with new infection medium for further 24 h. Cells were then grown in regular complete medium for 48 h and then selected by the addition of 5 μg/ml puromycin. The cells were then cultured until no more dying cells were observed and then transferred into T75 cell culture flasks with selection medium. PYHIN1 knockdown was assayed by qRT-PCR and immunoblot prior to the cells being used for experiments.

### Confocal microscopy of STING

NuLi-1 cells were seeded on a glass coverslip in 12-well plates and incubated overnight at 37 °C. Cells were then transfected with poly(dA-dT) and fixed 1.5, 3, or 6 h post transfection. The medium was removed and the cells fixed in cold methanol at −20 °C overnight. The next day the methanol was removed, the cells washed with PBS, the slides covered with a solution of 0.5% (v/v) Triton X-100 in PBS and incubated at room temperature for 12 min. Following further washing cells were blocked in 5% (v/v) FCS with 0.2% (v/v) Tween 20 in PBS for 1 h at room temperature. Next, anti-STING antibody was added (1:600 dilution) and the coverslips were incubated at 4 °C overnight. Cells were washed three times in PBS and fluorescently labeled secondary antibody (anti-rabbit IgG, Alexa Fluor 488 conjugated goat, Life Technologies) was added (1:1500 dilution in blocking buffer). Coverslips were then incubated for 3 h at room temperature in the dark. After three further washes, coverslips were mounted with the use of MOWIOL 4–88 containing 1 μg/ml DAPI. Images were then taken with the Confocal Laser Scanning Platform Leica TCS SP8 microscope.

### Statistical analysis

All data were analyzed with GraphPad Prism software. Statistical analysis was performed using unpaired Student's *t* test.

## Author contributions

D. M., M. B., and A. G. B. formal analysis; D. M. and A. G. B. investigation; D. M., M. B., and A. G. B. methodology; M. B., J. A. B., and A. G. B. supervision; M. B., J. A. B., and A. G. B. writing-original draft; J. A. B. and A. G. B. funding acquisition; A. G. B. conceptualization.

## Supplementary Material

Supporting Information

## Footnotes

2 The abbreviations used are:

PRR: pattern recognition receptor
cGAMP: cyclic GMP-AMP
cGAS: cyclic GMP-AMP synthase
ECACC: European Collection of Authenticated Cell Cultures
EV: empty vector
HSV: herpes simplex virus
MVA: modified vaccinia Ankara
NOD: nucleotide oligomerization domain
PAMP: pathogen associated molecular pattern
PMA: phorbol 12-myristate 13-acetate
PYD: pyrin domain
SeV: Sendai virus
TLR: Toll-like receptor.
