# Supplementary material for: PYHIN1 regulates pro-inflammatory cytokine induction rather than innate immune DNA sensing in airway epithelial cells
Source: J Biol Chem. 2020 Feb 26;295(14):4438–50. doi: 10.1074/jbc.RA119.011400 (PMC7135979; doi:10.1074/jbc.RA119.011400)
Supplement: Supporting Information [file supp_295_14_4438__index.html]

PYHIN1 regulates pro-inflammatory cytokine induction rather than innate immune DNA sensing in airway epithelial cells — PYHIN1 regulates inflammatory cytokine induction — PYHIN1 regulates pro-inflammatory cytokine induction rather than innate immune DNA sensing in airway epithelial cells — PYHIN1 regulates inflammatory cytokine induction — Supporting Information 

# PYHIN1 regulates pro-inflammatory cytokine induction rather than innate immune DNA sensing in airway epithelial cells

## Supporting Information

- Supporting Information (to be published online) - Figure S1
