# Supplementary material for: PYHIN1 regulates pro-inflammatory cytokine induction rather than innate immune DNA sensing in airway epithelial cells
Source: J Biol Chem. 2020 Feb 26;295(14):4438–50. doi: 10.1074/jbc.RA119.011400 (PMC7135979; doi:10.1074/jbc.RA119.011400)
Supplement: Supporting Information [file supp_RA119.011400_156344_2_supp_463844_q4kwpp.docx]

**
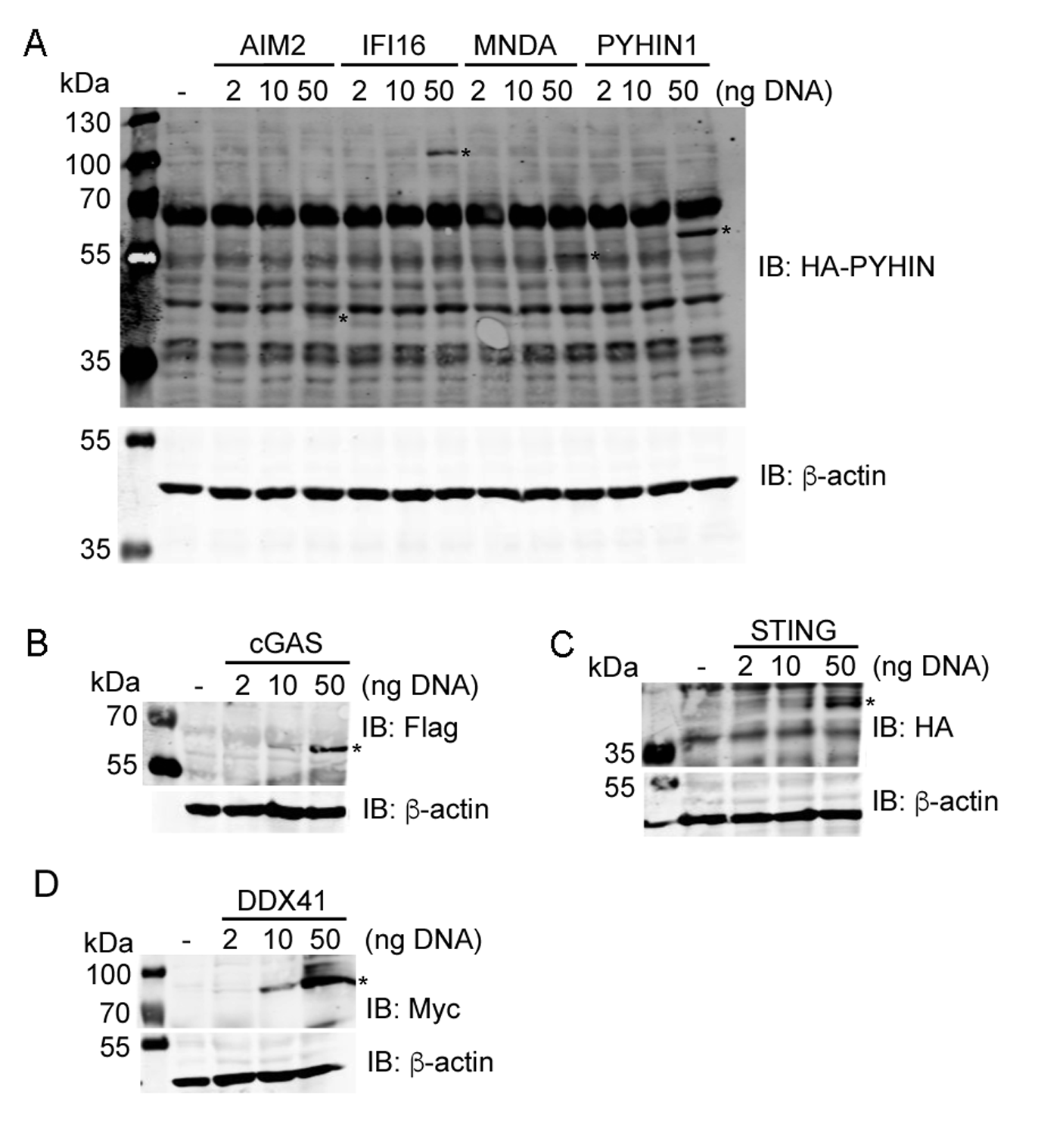
**

**Figure S1 (related to Figure 2). Expression of proteins encoded by transfected plasmids in A549 cells**

1x10^5^/mL A549 cells in 96 well plates were transfected with IFN-β promoter luciferase reporter together with empty vector or increasing amounts of AIM2, IFI16, MNDA, PYHIN1, cGAS, STING or DDX41 expression vectors exactly as per Figure 2. Wedges indicate increasing amount of the expression vectors (2, 10, 50 ng). Cells were lysed 24 h after transfection and assessed for protein expression by immunoblotting using anti-HA Ab to detect PYHINs (A), anti-Flag Ab to detect cGAS (B), anti-HA Ab to detect STING (C), anti-Myc Ab to detect DDX41 (D), and anti-β-actin Ab as a protein loading control (A-D). Asterisk (*) indicates expression of protein at the correct molecular weight. Immunoblots are representative of two experiments.
